# Survivin 2α: a novel Survivin splice variant expressed in human malignancies

**DOI:** 10.1186/1476-4598-4-11

**Published:** 2005-03-02

**Authors:** Hugo Caldas, Laura E Honsey, Rachel A Altura

**Affiliations:** 1 Center for Childhood Cancer, Columbus Children's Research Institute Columbus, OH, USA; 2 Department of Pediatrics, The Ohio State University, Columbus, OH, USA

## Abstract

**Background:**

Survivin and its alternative splice forms are involved in critical cellular processes, including cell division and programmed cell death. Survivin is expressed in the majority of human cancers, but minimally in differentiated normal tissues. Expression levels correlate with tumor aggressiveness and resistance to therapy.

**Results:**

In the present study, we identify and characterize a novel survivin isoform that we designate survivin 2α. Structurally, the transcript consists of 2 exons: exon 1 and exon 2, as well as a 3' 197 bp region of intron 2. Acquisition of a new in-frame stop codon within intron 2 results in an open reading frame of 225 nucleotides, predicting a truncated 74 amino acid protein. Survivin 2α is expressed at high levels in several malignant cell lines and primary tumors. Functional assays show that survivin 2α attenuates the anti-apoptotic activity of survivin. Subcellular localization and immunoprecipitation of survivin 2α suggests a physical interaction with survivin.

**Conclusion:**

We characterized a novel survivin splice variant that we designated survivin 2α. We hypothesize that survivin 2α can alter the anti-apoptotic functions of survivin in malignant cells. Thus survivin 2α may be useful as a therapeutic tool in sensitizing chemoresistant tumor cells to chemotherapy.

## Background

Alternative splicing is estimated to occur in 40–60% of all human genes, accounting for some of the discrepancies between the large number of known proteins and the three-fold lower number of human genes in the genome. Alternative splicing generates a multitude of isoforms that have overlapping but distinct functions during embryonic development and that also contribute to maintaining homeostasis in adult differentiated tissues (reviewed in [1]). Alternative splice forms of key proteins in cancer, TP53, MDM2 [2] and c-MYC [3], have been shown to play a role in oncogenesis.

**Figure 1:**
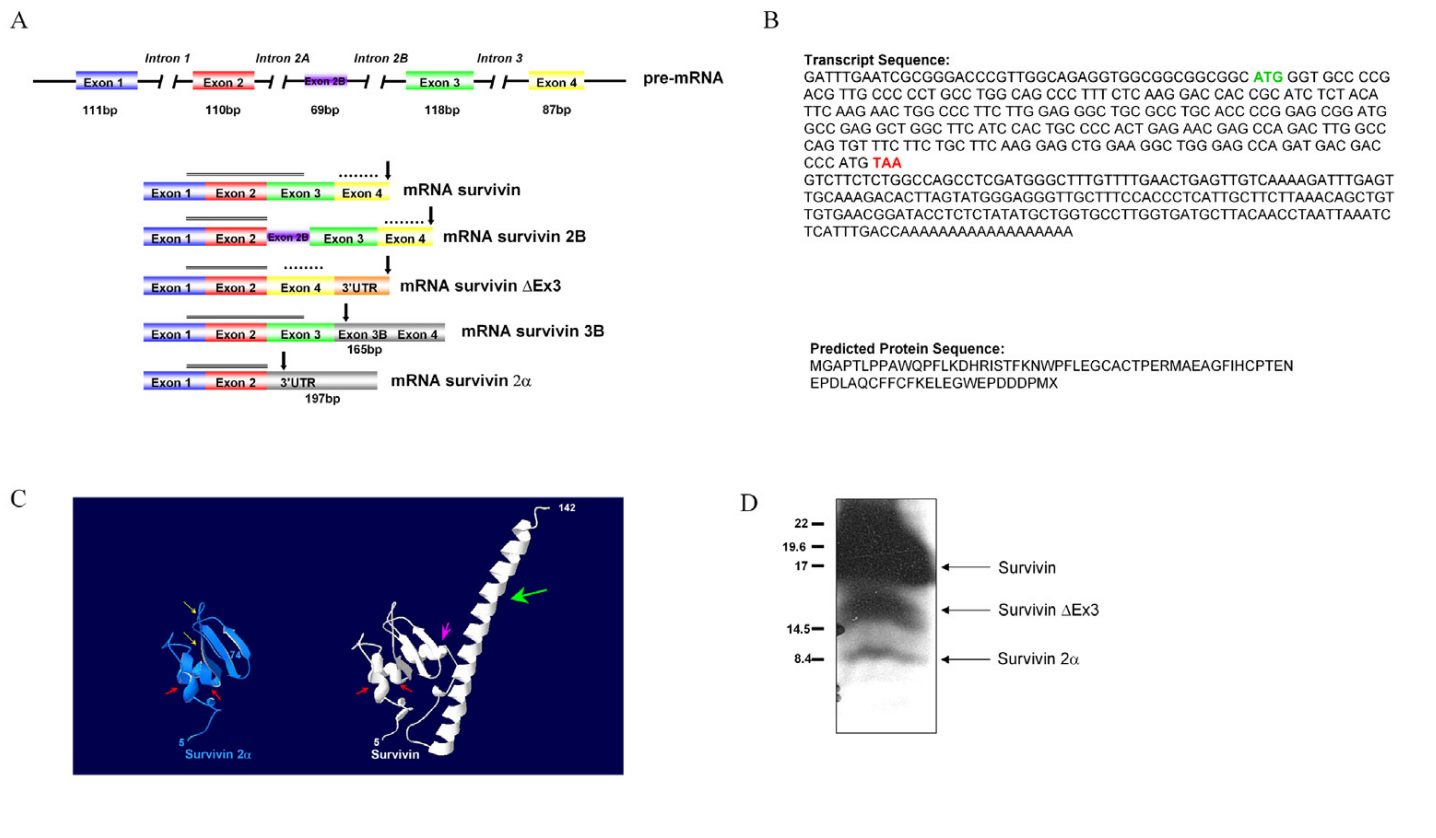
Structural analysis of survivin 2α compared with the other human survivin isoforms. A: The survivin pre-mRNA generates at least five mature mRNA transcripts. Boxes represent exons, with the sizes indicated below. The size of the additional nucleotide sequences for survivin 3B and survivin 2α are shown. In survivin 2α, 197nt of intron 2 are added, of which 195nt are non-coding. Protein domains and motifs are indicated above the diagrams. The black arrow indicates the stop codon; the coiled-coil domain is shown by the dotted line; and the double lines represent the BIR domain. B: Survivin 2α transcript and predicted protein sequence. The sequence for the survivin 2α transcript was obtained by sequencing the insert contained within the IMAGE clone. C: Predicted 3D structural modeling of survivin 2α and survivin. The amino acid sequence of survivin 2α was used with SWISS-MODEL to predict a 3D structure by homology modeling. The resulting PDB file was visualized and manipulated using Swiss PBD-Viewer for the view presented. The 3D structure for survivin was obtained from Swiss-PROT database (PDB entry 1F3H). The yellow arrows indicate regions of differences between survivin 2α and survivin. The red arrows represent the first 2 alpha helices of the BIR domain, and the pink arrow represents the 3^rd ^helix, that is absent in survivin 2α. The green arrow points to the C-terminal coiled-coil domain. D: Protein Analysis of survivin 2α. Total HeLa cell lysate was loaded on a 18% SDS-PAGE and transferred into nitrocellulose membrane. The proteins were detected by immunoprobing with a polyclonal survivin antibody. A protein of approximate molecular weight 8.5 kDa, corresponding to the predicted size of survivin 2α is detected in HeLa cell lysates.

Survivin was originally identified by structural homology to IAPs in human B-cell lymphoma [4]. It is composed of a single BIR domain and an extended carboxy-terminal coiled coil domain [5]. Transcription from the *Survivin *locus gives rise to alternatively spliced transcripts identified in both human and mice [6-8]. To date, three alternatively spliced isoforms have been described in humans [6-8]. Survivin-2B is generated by the insertion of an alternative exon, exon 2B; Survivin-Δ Ex3 arises from the removal of exon 3 resulting in a frameshift and translation of part of the 3'UTR generating a unique carboxy-terminus; Survivin-3B results from the introduction of a novel exon 3B resulting in a frameshift and premature termination of the protein (Figure 1A).

Survivin has 2 main functions; one as a chromosomal passenger protein [9] and the other as an inhibitor of apoptosis [10]. Survivin 2B has been shown to be a pro-apoptotic protein that sensitizes resistant leukemia cells to chemotherapy in a p53-dependent fashion [11]. Survivin-Δ Ex3 functions as an anti-apoptotic protein and is upregulated in malignancies (Mahotka et al., 1999). No function has yet been described for survivin-3B.

In this report we identify and characterize a novel isoform of survivin, survivin 2α. We show that survivin 2α is expressed at high levels in malignant cells, co-localizes with survivin and has the potential to attenuate the anti-apoptotic effect of survivin.

## Results and Discussion

### Structural Characteristics of Survivin 2α

In this work, we characterized a novel isoform of the survivin locus. We surveyed the aligned survivin EST sequences available in the UCSC Human Genome Browser and identified an EST from a human breast tumor cDNA library (I.M.A.G.E. clone 1631662). We sequenced the entire cDNA and designated it Survivin 2α. The complete cDNA sequence is shown in Figure 1B. The protein contains the coding sequences from exon 1 and exon 2, and one additional amino acid before termination (Figure 1B). This 74 amino acid protein, with a predicted molecular weight of 8.5 kDa, contains a truncated BIR domain and lacks the carboxy-terminal coiled-coil domain in its entirety (Figure 1A). There are no defined localization signals in the protein, and PSORTII predicts localization within the nucleus and the cytoplasm (Table 1). Alignment with the known human survivin isoforms shows that the sequence of Survivin 2α is identical to exons 1 and 2 of the other survivin splice variants, with the exception of the last amino acid. Alignment of Survivin 2α with the three mouse survivin isoforms also reveals some similarity with survivin40, a 40-amino acid mouse splice variant (not shown). The 3D predicted structure of Survivin 2α shows the absence of the alpha-helical coiled-coil domain, present in survivin (Figure 1C). It also shows minor predicted rearrangements in the structure that may occur to stabilize the protein (Figure 1C, yellow arrows). These re-arrangements occur within the BIR domain, and could have functional implications for the role of Survivin 2α in apoptosis.

**Table 1:** Table of the predicted localization and structural features of survivin and the novel isoform survivin 2α.

**Localization**	**Survivin**	**Survivin 2α**
Cytoplasm	56.5%	39.1%
Nucleus	17.4%	34.8%
Cytoskeleton	0%	4.3%
Golgi Apparatus	0%	0%
Plasma membrane	4.3%	0%
ER	4.3%	4.3%
Peroxysome	0%	0%
Mitochondria	13.0%	13.0%
Lysosomes	4.3%	4.3%

**Features**		

BIR	1	Partial
Coiled-Coil	1	0
Protein Size	142 aa	74 aa
Predicted Molecular Weight	16.4 kDa	8.5 kDa

The BIR domain has been shown to be important for homodimerization and coordination of the zinc atom co-factor [12]. In the survivin protein, Histidine 80 (H80) is required for zinc atom coordination and homodimerization. Expression constructs containing mutations at this residue within the Survivin protein have previously been shown to accelerate PCD (Programmed Cell Death) in vitro. Similarly, mutations in Cytosine 84 (C84) enhance PCD, as a result of displacement of the wild type Survivin protein [13]. The Survivin 2α protein, truncated at amino acid 74, lacks both of these amino acid residues. Additionally, Survivin 2α lacks the third alpha helix in the BIR domain. As the anti-apoptotic function of Survivin is mediated both by the BIR domain and by the interaction of its C-terminal coiled coil domain with microtubules of the mitotic spindle [10,14,15], it would be predicted that Survivin 2α might not have anti-apoptotic properties.

### Survivin 2α is highly expressed in tumor cells

Survivin is critical for global normal embryonic development, as demonstrated by the early embryonic lethality of mice with homozygous deletions in the survivin gene locus [16]. Survivin proteins are virtually absent from most normal differentiated tissues, however these proteins are expressed in certain highly proliferative areas within normal tissues [17-19]. In contrast, survivin is highly expressed in the majority of human malignancies, derived from different cell origins. We evaluated the expression of survivin 2α in 7 different cancer cell lines, 2 non-transformed tissues and 7 primary medulloblastoma tumors by quantitative PCR. We designed primers that will specifically amplify Survivin 2α after selection of polyadenylated RNA. Survivin 2α expression in tumor cells and primary medulloblastoma tumors varied from 2–100 fold above non transformed cells (Table 2). The levels of Survivin 2α transcripts are comparable to those of Survivin ΔExon3 (Table 3). Like Survivin, Survivin 2α is expressed at increased levels in transformed cells compared to non-transformed cells, and therefore it suggests that it could have a role in tumorigenesis. Additionally, we detected expression of endogenous Survivin 2α protein in HeLa cells, suggesting that the transcript is translated (Figure 1D).

**Table 2:** Survivin 2α expression (relative to normal tissue).

**Cell Type**	**Relative Increase**
Normal Cerebellum	1.00
Normal Breast (MCF10A)	0.97
Breast Carcinoma (MCF7)	8.17
Osteosarcoma (U2OS)	39.06
Lung (A549)	3.03
ALL (Jurkat)	1.84
Soft Tissue Sarcoma (RH28)	94.90
Cervical Carcinoma (HeLa)	58.22
Medulloblastoma (Daoy)	34.23

**Primary Tumors**	

Medulloblastoma #1	4.68
Medulloblastoma #2	154.55
Medulloblastoma #3	93.24
Medulloblastoma #4	5.69
Medulloblastoma #5	8.54
Medulloblastoma #6	9.81
Medulloblastoma#7	75.10

**Table 3:** Expression of survivin splice variants in medulloblastoma (relative to survivin)

**Tissue**	**Survivin 2B**	**Survivin ΔEx3**	**Survivin 2α**
Medulloblastoma #3	37.63	0.46	0.36
Medulloblastoma #4	43.82	0.18	0.18
Medulloblastoma (Daoy)	1290.16	35.10	0.66

### Functional Properties of Survivin 2α

**Figure 2:**
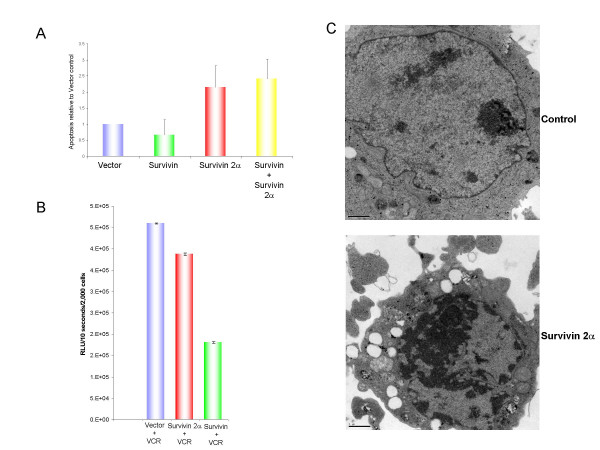
Early apoptosis in tumor cells transfected with survivin and survivin 2α. A: Daoy cells were transiently transfected with control vector, survivin, survivin 2α, or a combination of survivin and survivin 2α. Apoptosis was measured using Annexin V/PI staining 24 hours after transfection, in the absence of a cell death stimulus. The results are shown relative to empty vector control. Error bars represent standard deviations from triplicate experiments. Results were adjusted for transfection efficiency based on parallel transfection with a GFP-expressing plasmid. B: Daoy cells were transiently transfected with control, survivin or survivin 2α. Apoptosis was measured by Caspase-3 activity following treatment with vincristine. C: HeLa cells were transiently transfected with control or GFP-tagged survivin 2α. Electron Microscopy analysis of transfected cells shows a representative cell undergoing apoptosis as induced by survivin 2α. Images were taken 12,000× magnification and scale bars are shown.

To characterize a function for Survivin 2α, we transfected Daoy cells with Survivin 2α and a combination of Survivin 2α and Survivin. To induce apoptosis in the Daoy cells, we treated them with 2 μM of the chemotherapeutic agent vincristine. Vincristine is a vinca alkaloid that binds to tubulin, inhibiting microtubule polymerization. It kills Daoy cells in culture by PCD. We analyzed early apoptotic events in vincristine-treated transfected cells by Annexin V staining. Survivin 2α antagonized the anti-apoptotic effect of Survivin in co-transfection assays with or without a cell death stimulus (not shown and Figure 2A). As inhibition of apoptosis by Survivin involves activation of the caspase pathway [20], we assayed Survivin 2α transfected cells for caspase 3 activation. Caspase-3 was strongly activated in vector control and Survivin 2α transfected cells in the presence of vincristine. Much lower levels of caspase-3 activation were observed in Survivin-transfected cells (Figure 2B). In the absence of an apoptotic stimulus we observed a 35% increase of caspase-3 activity in Survivin 2α cells, as well as a 46% increase in early apoptosis, as assessed by Annexin V staining. We also performed electron microscopy analysis of Survivin 2α transfected and non-transfected cells. We sorted transfected cells from non-transfected cells by FACS based on GFP fluorescence, and processed each population for EM analysis (Figure 2C). Overall, there was a 43% increase in incidence of apoptosis in Survivin 2α-expressing cells versus non-expressing cells. Our results suggest that Survivin 2α can attenuate survivin's anti-apoptotic activity and sensitize tumor cells to chemotherapy. These findings have important therapeutic implications in the treatment of chemoresistant tumors.

### Survivin 2α alters the subcellular localization of survivin

**Figure 3:**
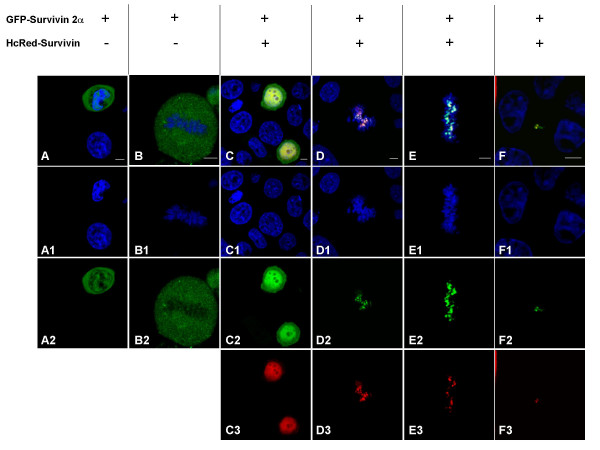
Confocal microscopy analysis of survivin 2α sub-cellular localization. HeLa cells were transfected with GFP-survivin 2α or HcRed survivin, as detailed on the top of the figure. Green pixels correspond to GFP expression, red pixels correspond to HcRed expression and blue pixels represent DNA labeled with Hoechst dye. When co-localization of GFP and HcRed occurs the pixels are yellow. A: Expression of survivin 2α at interphase localizes to nuclear and cytoplasmic structures. B: During M-phase survivin 2α is excluded from the condensed/dividing chromosomes and is localized in the cytoplasm of the dividing cell. C, D, E, F: When co-expressed with survivin, survivin 2α localization does not change at interphase. During M-phase survivin 2α co-localizes with survivin to the centromeres of the dividing chromosomes (D and E), and in the midbody region at cytokinesis (F). Scale bar = 5 μm

To characterize the subcellular localization of survivin 2α we performed direct fluorescence assays in HeLa cells transfected with a GFP- survivin 2α construct. Survivin 2α localized to the nucleus and the cytoplasm in interphase cells (Figure 3A). In cells undergoing mitosis, survivin 2α was confined to the cytoplasmic compartment (Figure 3B). Interestingly, when co-expressed with survivin, survivin 2α co-localized with survivin to the centromeres of the chromosomes in prometaphase (Figure 3C) and metaphase (Figure 3D), and at the midbody during late telophase/cytokinesis (Figure 3E). Moreover, the normal cytoplasmic localization of survivin shifted to the nucleus in interphase cells. This data suggests a direct interaction between the two proteins, as well as a potential mechanism for the attenuation of survivin's anti-apoptotic activity by survivin 2α.

### Survivin 2α physically interacts with survivin

**Figure 4:**
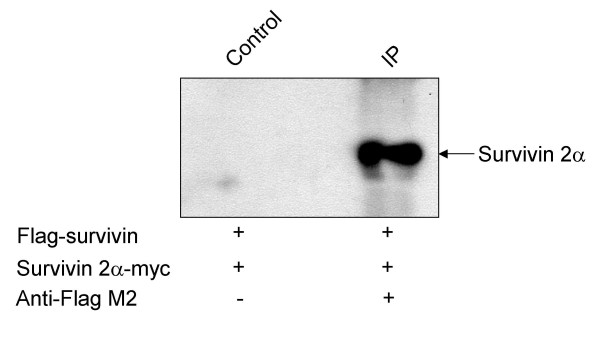
Co-immunoprecipitation of survivin-survivin 2α. HeLa cells were transfected with constructs encoding tagged forms of survivin (Flag) and survivin 2α (myc). Lysates from transfected cells were subjected to immunoprecipitation with an antibody against the Flag epitope. The resulting immunoprecipitated complexes were resolved by SDS-PAGE and subjected to Western blotting. The membrane was immunoprobed with an antibody against the myc epitope tag. Survivin 2α-myc is clearly visualized in lysates precipitated with a Flag antibody.

To further investigate the possibility that survivin 2α interacts with survivin we performed co-immunoprecipitation experiments. We co-transfected HeLa cells with constructs encoding a Flag-survivin fusion protein and a myc-survivin 2α fusion protein. We used a Flag antibody to precipitate protein complexes, and a myc antibody to detect myc-tagged survivin 2α. We detected survivin 2α-myc in the complexes precipitated with the Flag antibody, substantiating a physical interaction of survivin with survivin 2α (Figure 4).

## Conclusion

We characterized a novel survivin splice variant that we designated survivin 2α. We hypothesize that survivin 2α can alter the anti-apoptotic functions of survivin in malignant cells. Thus, survivin 2α may be useful as a therapeutic tool in sensitizing chemoresistant tumor cells to chemotherapy.

## Methods

### Patient samples

Seven fresh frozen primary medulloblastoma tumor samples were obtained from the Cooperative Human Tumor Network (CHTN), after approval through the Columbus Children's Hospital IRB.

### Sequencing

IMAGE clone 1631662 (Invitrogen) was sequenced using primers that flanked the multiple-cloning-site.

### Plasmids and Cloning

The cDNA for survivin 2α was amplified from the EST clone (Invitrogen) and cloned into the *Kpn*I-*Bam*HI sites of pcDNA4/TO/myc-HisB (Invitrogen) generating an in-frame fusion with the C-terminal myc-tag, or into the *Kpn*I-*Bam*HI sites of pEGFP-N3 generating an in-frame fusion with the C-terminal GFP tag. The start codon in both constructs corresponds to the naturally occurring start codon in the cDNA transcript. The resulting clones were confirmed by sequencing.

### Cell Culture and Transfection

HeLa (cervical adenocarcinoma), Daoy (medulloblastoma), Jurkat (acute lymphoblastic leukemia) and MCF-7 (breast adenocarcinoma) cells (ATCC) were grown in DMEM supplemented with 10% FBS at 37°C, 5% CO_2_; U2OS osteosarcoma cells (kindly donated by Dr. Greg Otterson) were grown in McCoy's 5A medium supplemented with 10% FBS at 37°C, 5% CO_2_; RH28 (alveolar rhabdomyosarcoma, kindly donated by Dr. Steve Qualman) and A549 (lung carcinoma) (ATCC) were grown in RPMI1640 supplemented with 10% FBS at 37°C, 5% CO_2_. MCF10-A, a non-transformed breast cell line (ATCC) was grown in MEGM, Mammary Epithelial Growth Medium, Serum-free, (Clonetics) supplemented with 100 ng/ml cholera toxin (Sigma Aldrich) at 37°C, 5% CO_2_.

Transient transfections were performed using Effectene transfection reagent (Qiagen) at a DNA: Effectene ratio of 1:10.

### Drug Treatment

Induction of apoptosis by vincristine was done by treatment of cells with complete growth medium supplemented with vincristine sulfate at a final concentration of 2 μM.

### RNA isolation and Real Time PCR

RNA was isolated from 10^6 ^proliferating cells or frozen tumor tissue using TriZol reagent (Invitrogen) as recommended by the supplier. Poly(A) RNA was purified using Oligotex dT kit (Qiagen). 100 ng of poly(A) purified RNA was used as a template in a reverse transcription reaction using random hexamers and Omniscript Reverse transcriptase (Qiagen) and performed according to manufacturer's instructions. Quantitative real-time PCR reactions using Taqman probes (FAM/TAMRA) were run in triplicate on an ABI Prism 7700 Real-time PCR machine (Applied Biosystems). Control GAPDH reactions (Applied Biosystems) were run to normalize ΔCt values. Relative change was calculated by the comparative C_T _method, 2^(-ΔΔCt)^. The survivin 2α specific primers consist of: Forward 5'GCTTTGTTTTGAACTGAGTTGTCAA; Reverse 5'GCAATGAGGGTGGAAAGCA; and Probe: 6FAM AGATTTGAGTTGCAAAGACACTTAGTATGGGAGGG TAMRA

### Apoptosis Assays

Two apoptosis assays were performed: Caspase-3 assay and Annexin-V FLUOS. For caspase assays 2,000 cells from each experimental condition were subjected to the caspase-3 assay, Caspase 3/7 GLO (Promega) and analyzed on a Victor3 plate reader (Applied Biosystems). Experiments were performed in triplicate.

Annexin V/propidium iodide staining was carried out using the Roche Annexin-V-Fluos Staining Kit following the manufacturer's instructions. Fluorescein and propidium iodide fluorescence measured with a Coulter EPICS XL flow cytometer. Experiments were performed in triplicate.

### Microscopy

Proliferating HeLa cells, grown on glass coverslips, were transiently transfected with a GFP-tagged survivin 2α expression construct or co-transfected with GFP-tagged survivin 2α and HcRed-tagged survivin. 24 hours post-transfection the cells were fixed in 4% paraformaldehyde and stained with 50 μg/ml Hoechst dye. Cells were analyzed on a Zeiss LSM510 META confocal microscope, using a 63x PlanApochromat objective. For electron microscopy analysis, proliferating HeLa cells were transfected with GFP-tagged survivin 2α construct for 12 hours. The cells were aseptically sorted by FACS based on green fluorescence from GFP-survivin 2α for positive and negative populations. This was done in order to separate an enriched population that consisted of >90% GFP expressing cells. 10^6 ^cells for each condition were fixed in 2.5% gluteraldehyde for 24 hours and processed for EM. For cell analysis, 10 to 12 fields containing 8–10 cells per field at a magnification of 3500× were used. At least 100 cells were counted for each experimental condition and assigned to categories of healthy or dying based on their morphological appearance, including nuclear integrity. Image collection was performed on a Hitachi H-600 transmission electron microscope equipped with a GATAN image acquisition system.

### Co-Immunoprecipitation

HeLa cells transfected with Flag-survivin and survivin-2α myc were collected in Cell Lysis Buffer (100 mM Tris-HCl pH8.0, 100 mM NaCl, 0.5% Triton X-100, 0.2 μM PMSF) and incubated at 4°C for 30 min. The cell lysate was clarified by centrifugation and the clarified supernatant dissolved 1:5 in Co-IP buffer (50 mM Tris-HCl pH7.5, 15 mM EGTA, 100 mM NaCl, 0.1% Triton X-100, 1x protease inhibitors cocktail, 1 mM DTT, 1 mM PMSF). The equivalent of 400 μg of lysate total protein was incubated with 2 μg of anti-Flag M2 antibody at 4°C for 1 h with constant rotation. As a control the same amount of lysate protein was incubated in the absence of antibody. Fifty microliters of agarose-conjugated protein A (Invitrogen) were added and the mixture incubated for a further hour in the same conditions. The protein-antibody-protein A complexes were pulled down by centrifugation and subjected to 3 washes with co-IP buffer. The proteins were analyzed through electrophoretic separation in a 20% SDS-PAGE, electroblotted onto nitrocellulose and immunoprobed with an antibody against myc-tag. Detection was performed using the ECL kit (Amersham). Protein standards were used for size determination.

### Bioinformatics

Subcellular localization predicted by PSORTII program. Coiled-Coil domain predicted by Coils and PairCoil programs

## Authors' contributions

HC performed bioinformatic analysis, subcellular localization, functional studies, co-immunoprecipitation and drafted the manuscript. LH performed quantitative real time PCR in cell lines and primary tumors. RA conceived the study and participated in its design and coordination, and was responsible for overseeing the final version of the manuscript. All authors have read and approved the final manuscript.
